# A controversial change in the sustainable development goal indicator for access to medicines

**DOI:** 10.2471/BLT.25.294862

**Published:** 2026-05-11

**Authors:** Iris R Joosse, Laura Doblas Rodriguez de Guzman, Aukje K Mantel-Teeuwisse, Hendrika A van den Ham

**Affiliations:** a Utrecht Institute for Pharmaceutical Sciences, Utrecht University, Heidelberglaan 8, 3584 CS Utrecht, Kingdom of the Netherlands.

Medicines are a fundamental building block of effective health systems, yet data consistently show that access to essential medicines remains inadequate.^1^ Monitoring how well health systems provide access to medicines is essential for shaping national and international political priority-setting and developing targeted interventions.^2^ Since 2018, this critical building block has been captured in sustainable development goal (SDG) indicator 3.b.3, which measures the proportion of health facilities that have a core set of essential medicines available and affordable. Here, we express our regret that in March 2025, the Inter-Agency and Expert Group on Sustainable Development Goal Indicators decided to intervene before the SDG agenda had run its course and replace this critical indicator with a new health product access index.^3^ We strongly advocate for renewed commitment to existing methods for monitoring access to essential medicines in addition to the new index, amplifying previous warnings about the future of monitoring access.^4^^,^^5^

## The road to replacement

The Inter-Agency and Expert Group on Sustainable Development Goal Indicators made the decision to replace the indicator during the 2025 Comprehensive Review Process of the global indicator framework.^6^ Leading up to this decision, the World Health Organization (WHO) had submitted a replacement proposal in April 2024, outlining the intention to replace the indicator with the new index. The proposal was subsequently included in the global open consultation held mid-2024, a period when many countries have extended holiday breaks, which potentially limited engagement of key stakeholders and experts. Most of the relatively few comments submitted during the consultation round supported replacing the existing indicator; however, the respondents also raised multiple critical remarks to the proposed replacement. Despite these concerns, some of which highlighted substantive issues, the Inter-Agency and Expert Group on Sustainable Development Goal Indicators eventually accepted the proposal without changes.

## Grounds for replacement

A key justification for replacing SDG indicator 3.b.3 was the limited data coverage. Currently, the WHO database includes information from only 25 countries for this indicator, with the most recent data predating 2020.^7^ The laborious nature of data collection at health facilities may explain this data gap and be the reason why many low- and middle-income countries struggle to prioritize and allocate adequate budgets to these surveys. However, a recent study in Albania, mapping the time, human and financial resources needed to conduct such a survey suggests that the required investments are both feasible and justifiable.^8^

Rather than eliminating the indicator, efforts should have focused on making data collection more efficient. Advancements in electronic data collection and the use of sentinel-site monitoring, where selected health facilities regularly report data, offer promising alternatives. The latter approach, already used in health surveillance and pharmacovigilance, remains unexplored in monitoring access to medicines. This approach could mobilize pharmacists and other health workers as data collectors and enhance the reliability and frequency of reporting. Additionally, data collection for medicines could be integrated into other existing routine monitoring systems, which also depend on data collection at health facility level and sentinel-site monitoring.^9^ Data collected by academic research groups are an opportunity for innovative data collection systems but also remain an untapped resource, which countries and the United Nations have yet to leverage effectively.^7^

Another argument supporting the elimination of indicator 3.b.3 concerned the use of disease burden weighting in calculating access,^10^ which may not accurately account for regional variations in disease burden and medication use. This weighting step was designed to make the indicator more relevant for countries with a low burden of infectious diseases, such as human immunodeficiency virus, malaria and tuberculosis, for which relevant medicines were included in the indicator. However, instead of correcting differences in disease burden through the weighting step, the medicines with very low national relevance can also be removed from analysis altogether, thereby also reducing the number of medicines for which data need to be collected in a survey. Recent analyses in an Albanian case study demonstrated that these approaches indeed yield nearly identical results (Joosse et al., Utrecht University, unpublished data, 24 April 2026). Although this analysis was not repeated in countries with a high burden of these infectious diseases, the weighting step added limited value in these settings as all of the medicines included in the indicator are by definition essential in these countries.^10^ In conclusion, removal of the weighting step would considerably simplify use of the technically complex and data-intensive indicator,^4^ without replacing it.

Other known limitations of the original indicator, which captured both availability and affordability of medicines, relate to its binary design (1 meaning present on the day of data collection or 0 meaning not present on the day of data collection).^10^ As a result, the indicator failed to capture the volume of medicines needed to meet actual demand. Additionally, the indicator defined affordability using the daily wage of the lowest-paid governmental worker. This approach may not reflect the income levels of large parts of the population, and it did not account for household-level factors such as dependence on a single income or the extent of health insurance coverage.

## The replacement

The new index removes the additional burden of data collection by relying solely on existing data sources and indicators that are already part of WHO’s established reporting system, unlike the original indicator.^6^ However, the key argument against the replacement of the indicator by the new index is its failure to address critical medicine needs. The new index relies on a combination of 19 health service coverage indicators, including nine measuring vaccination and immunization coverage but only three that directly relate to medicine access (coverage of tuberculosis treatment, antiretroviral therapies and reproductive health products).^3^

As the 19 indicators were selected primarily on the basis of their established use rather than their relevance, the resulting index does not achieve the appropriate balance between convenience and accuracy. For example, the index no longer captures the availability of medicines, at a time when shortages and stock-outs are becoming more frequent worldwide. Affordability is also omitted, even though it is a prerequisite for access to medicines. This omission is particularly concerning given recent interruptions in major donor funding flows for medicines such as antiretroviral therapies and antituberculosis medicines. Furthermore, medicines for noncommunicable diseases are almost completely disregarded in the new index,^5^ despite the clear need to increase access to these medicines^1^ and the global commitment to reduce the burden of noncommunicable diseases as captured in SDG target 3.4. Similarly, the specific needs of children remain overlooked,^11^ even though researchers suggested and validated a child-specific counterpart of the current SDG indicator 3.b.3.^10^ Meanwhile, vaccination and immunization coverage is now captured twice within the Global Indicator Framework, with SDG indicator 3.b.1 already measuring vaccine coverage.

A further concern with the health product access index is its failure to meet key criteria for a meaningful access indicator. According to a panel of 40 experts, indicators must be universal, relevant and actionable for policy-makers.^2^ An index that excludes noncommunicable disease medicines cannot be considered universal. Moreover, its complexity (since it combines19 health access components in a single index) risks making it difficult for policy-makers to interpret and act upon effectively. Of note, stakeholders already highlighted this concern during the Global Open Consultation.^6^

Another concern repeatedly raised during the consultation was the need to validate the proposed index before adopting it as a replacement, to ensure that it accurately reflects access to medicines and addresses the needs of policy-makers and other stakeholders. To our knowledge, WHO did not validate or test the new index before its adoption. After the Inter-Agency and Expert Group on Sustainable Development Goal Indicators adopted the original indicator, several years of piloting and technical refinement^8^ were required before all concerns were addressed. With only a few years remaining for SDG monitoring, replicating such a thorough process seems no longer feasible for the new index, which raises questions about the appropriateness of replacing the indicator at this late stage in the SDG timeline.

## Safeguarding access monitoring

Although the health product access index may generate useful insights into several aspects of health-care delivery, it fails to capture essential medicines adequately. We therefore call for the continued use of an indicator that has been validated and proven to provide comprehensive insights into access to medicines,^10^ rather than its complex replacement that risks overlooking one of the fundamental health system building blocks. The well-established WHO/Health Action International method for collecting the data, needed as input for SDG indicator 3.b.3, also remains a valuable option and such surveys should still be undertaken. Despite their known flaws,^10^^,^^12^ these existing methods provide information on a range of essential medicines that the new index does not.

The replacement of indicator 3.b.3 has already impeded ongoing efforts to collect critical data on medicine access, with the global community again being at risk of failing to report on this vital building block.^13^ As the SDG agenda moves towards its 2030 deadline, we urge policy-makers and researchers to continue to apply existing monitoring methods on access to medicines alongside the new index. At the same time, focus must shift beyond 2030. To ensure accurate monitoring of access to medicines in the future, WHO, governments and research groups should both intensify data collection on essential medicines and explore more efficient data collection approaches, such as sentinel-site monitoring. Additionally, definitions and indicators must be redefined to address the known limitations of existing monitoring tools.^12^ Without such efforts, WHO and the larger global health community risk repeating past mistakes, mainly the failure to report on access to medicines, and face yet another failed indicator on access to medicines in the years to come.
